# Ranking Metabolite Sets by Their Activity Levels

**DOI:** 10.3390/metabo11020103

**Published:** 2021-02-11

**Authors:** Karen McLuskey, Joe Wandy, Isabel Vincent, Justin J. J. van der Hooft, Simon Rogers, Karl Burgess, Rónán Daly

**Affiliations:** 1 Glasgow Polyomics, University of Glasgow, Glasgow G61 1QH, UK; karen.mcluskey@glasgow.ac.uk (K.M.); joe.wandy@glasgow.ac.uk (J.W.); 2 IBioIC, Strathclyde Institute of Pharmacy and Biomedical Sciences, University of Strathclyde, Glasgow G1 1XQ, UK; isabel.vincent@glasgow.ac.uk; 3 Bioinformatics Group, Wageningen University, 6708 PB Wageningen, The Netherlands; justin.vanderhooft@wur.nl; 4 School of Computing Science, University of Glasgow, Glasgow G12 8QQ, UK; simon.rogers@glasgow.ac.uk; 5 Centre for Synthetic and Systems Biology, School of Biological Sciences, University of Edinburgh, Edinburgh EH9 3JG, UK; Karl.Burgess@ed.ac.uk

**Keywords:** liquid chromatography–mass spectrometry (LC/MS), pathways, molecular family, Mass2Motif, SVD, matrix decomposition, metabolite sets

## Abstract

Related metabolites can be grouped into sets in many ways, e.g., by their participation in series of chemical reactions (forming metabolic pathways), or based on fragmentation spectral similarities or shared chemical substructures. Understanding how such metabolite sets change in relation to experimental factors can be incredibly useful in the interpretation and understanding of complex metabolomics data sets. However, many of the available tools that are used to perform this analysis are not entirely suitable for the analysis of untargeted metabolomics measurements. Here, we present PALS (Pathway Activity Level Scoring), a Python library, command line tool, and Web application that performs the ranking of significantly changing metabolite sets over different experimental conditions. The main algorithm in PALS is based on the pathway level analysis of gene expression (PLAGE) factorisation method and is denoted as mPLAGE (PLAGE for metabolomics). As an example of an application, PALS is used to analyse metabolites grouped as metabolic pathways and by shared tandem mass spectrometry fragmentation patterns. A comparison of mPLAGE with two other commonly used methods (overrepresentation analysis (ORA) and gene set enrichment analysis (GSEA)) is also given and reveals that mPLAGE is more robust to missing features and noisy data than the alternatives. As further examples, PALS is also applied to human African trypanosomiasis, Rhamnaceae, and American Gut Project data. In addition, normalisation can have a significant impact on pathway analysis results, and PALS offers a framework to further investigate this. PALS is freely available from our project Web site.

## 1. Introduction

An organism’s metabolism is comprised of all of the chemical reactions involved in its cells; the small-molecule intermediates and products of these reactions are known as metabolites. Untargeted metabolomics provides a profile of all detectable metabolites in a system, allowing the unique chemical fingerprint left behind by the metabolism to be investigated. Such studies are imperative in understanding changes in biological mechanisms related to environmental and genetic variations. Mass spectrometry (MS) is one of the most commonly used techniques for untargeted metabolomics and is often coupled with chromatographic separation using liquid chromatography (LC). Experimental data from a typical LC–MS experimental sample are a series of spectra, where signal intensities are generated for each detected ion. After data preprocessing, a table of chromatographic peaks is produced; each peak can be represented by its mass-to-charge ratio (m/z), retention time (RT), and intensity values. Peaks with a similar m/z and RT can then be grouped across samples into features, which leads to a matrix of peak intensities, indexed by features along the rows and samples as the columns. Identification is performed to associate features with metabolite identities, either by matching the m/z and RT of features with internal library standards or through fragmentation spectra comparisons against mass spectral databases.

In a cell or organism, metabolites seldom work alone. As result, it it often useful to analyse related groups of metabolites rather than considering individual metabolites in isolation. One way to define metabolite sets is through prior knowledge of how metabolites participate in a series of related chemical reactions, or metabolic pathways. Ranking biologically relevant pathways through changes in the intensities of associated features provides high-level information that helps prioritise relevant pathways [1]. An alternative way of producing metabolite sets is to to group features using spectral similarities, which can be incredibly useful for helping to identify unknown features. By exploiting the fragmentation spectra from tandem mass spectrometry, molecular networking [2] uses such spectral similarities to group metabolites into molecular families (MF). These MFs potentially correspond to chemical classes and have proved invaluable in enhancing the putative identifications of unknown metabolites [3,4]. Using tandem MS (MS2) and an algorithm originally used for text mining, latent Dirichlet allocation (LDA), MS2LDA [5] features are grouped by co-occurring fragments and neutral losses into Mass2Motifs that reveal potentially related chemical substructures.

In recent years, enrichment analysis algorithms, originally developed for analysing large collections of genes or transcripts, have been adapted to metabolomics. These methods were traditionally used to test gene expression data for functions or processes that are overrepresented (i.e., enriched) with regard to functional gene sets, pathways, and networks. Considering differentially expressed (DE) gene sets, two of the most commonly used types of gene enrichment techniques are overrepresentation analysis (ORA) and functional class scoring (FCS). ORA approaches rely on testing for the presence or absence of significantly changing genes in a pathway, using statistical significance tests. FCS methods, which include gene set enrichment analysis (GSEA) [6] use statistical approaches to identify gene sets that are significantly enriched or depleted in the pathway. FCS improves upon ORA by eliminating the need to preselect significantly changing genes, as well as taking into account how groups of genes can be coexpressed together (instead of assuming they are independent, as in ORA). One such FCS method is pathway level analysis of gene expression (PLAGE) [7], which performs singular value decomposition (SVD) to compute an activity value from expression data in a sample. This has advantages over other methods in that it is computationally simple while returning high performance in both sensitivity and specificity [8]. In addition, the PLAGE method does not discriminate between upward or downward activity, meaning that both increases and decreases in gene expression contribute to the activity of a pathway.

The nature of metabolomics data can make enrichment analysis for metabolite groups more challenging than for groups of genes. In particular, an untargeted metabolomics experiment contains a large diversity of chemical structures with the identity of many of the features unknown in mass spectral databases. In addition, there is uncertainty in annotating an MS feature, as a single compound can be matched to multiple features and vice versa even at a high mass accuracy of 1 ppm [9]. Other more general omics issues, such as missing data, also influence metabolite pathway reconstruction from metabolomics data. Finally, being the endpoint of the omics cascade, metabolite signals within a pathway may fluctuate or, as a result of a dead-end metabolic reaction, different features may change in opposite directions. As demonstrated in our previous work [5] where the PLAGE method was successfully applied to the enrichment analysis of Mass2Motifs, decomposing activity levels via SVD appeared to work well on metabolomics data where noise and missing data are prevalent. However, no comparisons were made in [5] to assess the performance and robustness of PLAGE against the alternatives, or to produce a general tool that could be used by the metabolomics community to assess the activity level of metabolite sets, including pathways. While a variety of tools that encompass enrichment analysis of pathways have been developed, many are Web-based [10,11,12] or implemented in the R programming language [13,14,15,16]. A comprehensive metabolite set analysis that can be easily integrated into Python-based workflows and applications remains rare.

In this manuscript, we introduce Pathway Activity Level Scoring (PALS), a comprehensive and modular Python tool for metabolite set enrichment. PALS is designed to look at how metabolic pathways or other groups of metabolites change between different experimental conditions. It achieves this by decomposing peak intensities into latent factors corresponding to observed metabolic changes (activities). Our adaptation of the PLAGE method, denoted as mPLAGE (PLAGE for metabolomics), was independently implemented in Python for use in metabolomics. Using mPLAGE, small correlated changes in a group of metabolites are rated as more interesting than large changes in an individual metabolite, where other metabolites in the set are not changing. The changes are increases and/or decreases in feature intensities, but must occur as part of the group. This is particularly important in situations where enzyme inhibition results in substantial increases to metabolite abundance before a lesion in a pathway, with concomitant decreases after the lesion (see [17] for an example). The modularity of PALS also means it is not limited to pathways, and any user-defined grouping of features (representing metabolite sets) can be readily analysed. This is demonstrated by assessing the activity levels of metabolite sets grouped by similarity of fragmentation spectra. The robustness of mPLAGE was evaluated on both synthetic and real data experiments and compared to both ORA and GSEA methods. The results demonstrated that the mPLAGE method is robust to noise and missing values, which is particularly important for metabolomics data. PALS is freely available at https://pals.glasgowcompbio.org/ (accessed on 5 February 2021) [18]. It can be easily imported as a Python library, run as a standalone tool or used as a Web application.

## 2. Results and Discussion

### 2.1. Synthetic Data Experiments

#### 2.1.1. Synthetic Data Setup

To assess the robustness of mPLAGE, benchmarking experiments were performed. Initially, these experiments were performed using synthetic data, as the ground-truth of untargeted metabolomics experiments can be hard to define. In this section, pathways are described for simplicity, but this procedure could be applied to any set of metabolites. Seven synthetic pathways were constructed with significant changes (showing clear block structures between the case and control groups in their peak intensity matrix) between two experimental conditions, each consisting of four samples. To mimic the inclusion of formulae that arise due to incorrect identification/annotation, decoy features were generated using a normal distribution (Supplementary Section S3) and standardised for input (Supplementary Section S1).

Each of the seven pathways contained a different number of formulae, and each pathway was named according to the number of features associated with it for clarity, i.e., pathway names for numbers of features were: Two; Four; Six; Ten; Twenty; Forty; and Eighty. A one-to-one relationship between an observed feature and a metabolite was assumed. In addition, 100 background pathways, where each pathway contains between 5 to 50 metabolites that show no significant changes between the case and control groups, were generated and added to the synthetic data set. To simulate missing features, which often occurs in real metabolomics data, features were also randomly removed from pathways with a uniform probability of 0.2. The performance of mPLAGE was compared to widely used overrepresentation (ORA) analysis and gene set enrichment analysis (GSEA) methods (described further in Supplementary Section S4). These alternative methods, used for benchmarking experiments, are included in the PALS Python library, allowing all methods to be compared utilising the same database query codes and retrieving identical pathways from KEGG and Reactome.

#### 2.1.2. Evaluation

The following evaluation metrics were implemented to assess the performance of the different methods. Let *T* = {Two, Four, Six, Ten, Twenty, Forty, Eighty} be the set of true answers (the labels of seven pathways with the corresponding number of features that are actually changing). Each pathway analysis method *m* will return $P_{m}$, a ranking of significantly changing pathways under some threshold α (set to 0.05 following convention), where pathways with *p*-values below α are considered significant (positives).

For a method *m*, the following counts can now be evaluated: True Positives (TP) = in $P_{m}$ and in *T*; False Positives (FP) = in $P_{m}$ but not in *T*; False Negatives (FN): in *T* but not in $P_{m}$. To obtain single-number summaries, precision and recall are used. Here, precision (Prec=TP/(TP+FP)) measures pathway ranking relevancy and it often occurs as a trade-off to recall (Rec=TP/(TP+FN)), which measures how many actual relevant changing pathways are returned. High precision suggests that a method has a low false positive rate, while a high recall suggests that the method has a low false negative rate. To summarise overall performance, the $F_{1}$ score, which is a harmonic mean of precision and recall and defined as $F_{1}=\left(2*Prec*Rec\right)/\left(Prec+Rec\right)$, is used.

#### 2.1.3. Synthetic Experiment Results—Increasing the Number of Decoy Features

In this experiment, an increasing number of decoy features were added to the seven pathways comprised of significantly changing features. This scenario reflects the case of attempting to find pathways, or sets of metabolites, with high activity levels when the number of significantly changing metabolites are small compared to noisy data, nonchanging or wrongly annotated metabolites. The level of decoy features are defined in an increasing order of severity from 0%, 25%, 50%, 100%, 250%, 500%, and 1000% of the original number of features in the pathway. For example, adding 50% decoy features to pathway Forty results in the 40 significantly changing features and the addition of 20 new decoy (nonchanging) features in the pathway. The resulting synthetic data matrix with the addition of decoy features is then used as input to the methods being compared. This procedure is repeated 500 times for each level of decoy features.

**Figure 1:**
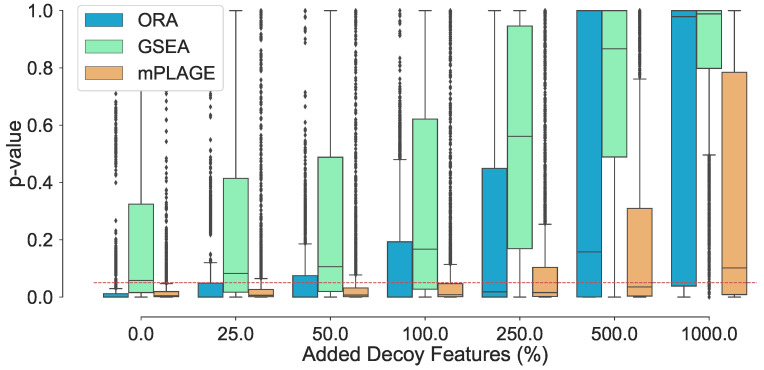
Adding nonchanging decoy features to the seven known changing pathways T={2,4,6,10,20,40,80}. The percentage of decoy features represents the number of additional noisy features added to a pathway as a percentage of the original number of features in that pathway. The boxplots show the spread of the *p*-values calculated from the pathways, including the median (solid horizontal line). The red dashed line indicates a *p*-value threshold of 0.05. The results show that increasing noise levels generally produces higher *p*-values, making it harder to detect significantly changing pathways. mPLAGE is more robust compared to other methods in returning lower *p*-values in the presence of noise.

Assessing changes to the *p*-values of the true changing pathways with higher number of decoy features reveals that increasing the number of decoy features generally produces higher *p*-values (Figure 1). This suggests that it becomes harder for pathways to be identified as significantly changing when a greater number of decoy features are present. At level 0% (where no additional decoy features are present), both ORA and mPLAGE perform well, returning median *p*-values of $2\times 10^{-6}$ and 0.0049, respectively. GSEA performs less well with median *p*-value of 0.0578, which is close to the selected significant threshold of 0.05 (shown as dotted line in Figure 1). The mean *p*-values from all methods increase as the number of decoy features is increased. At level 100%, both ORA and mPLAGE perform well even when there are as many decoy features as there are actually changing features, returning median *p*-values of 0.0083 and 0.0003 for mPLAGE and ORA, respectively. At higher levels (250%, 500%, and 1000%), GSEA appears most sensitive to the perturbation, while mPLAGE performs best amongst the methods compared.

**Figure 2:**
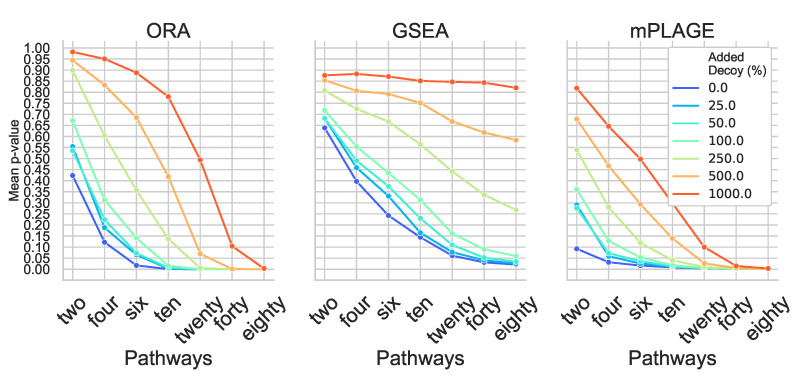
Mean *p*-values from overrepresentation analysis (ORA), gene set enrichment analysis (GSEA), and pathway level analysis of gene expression for metabolomics (mPLAGE) calculated for each significantly changing synthetic pathway at different levels of decoy features added. Across all levels of added decoy features, it is easier to identify larger pathways as significantly changing than smaller pathways. mPLAGE generally returns lower *p*-values than ORA and GSEA.

Inspection of individual significant pathways of varying sizes (Figure 2) revealed that the mean *p*-values returned by all methods generally decreases with an increasing number of changing features in a pathway. This suggests that the greater the number of metabolites that are changing together, the higher ranked that pathway would be. As a result of the small number of metabolites in the pathway, all methods struggle to correctly identify pathways Two and Four as significantly changing at any level of added decoy features and generally perform better on the larger pathways Twenty, Forty, and Eighty. This reveals that the larger the number of changing features identified in a pathway (or metabolite set), the more tolerant the method is to nonchanging (random) features.

**Figure 3:**
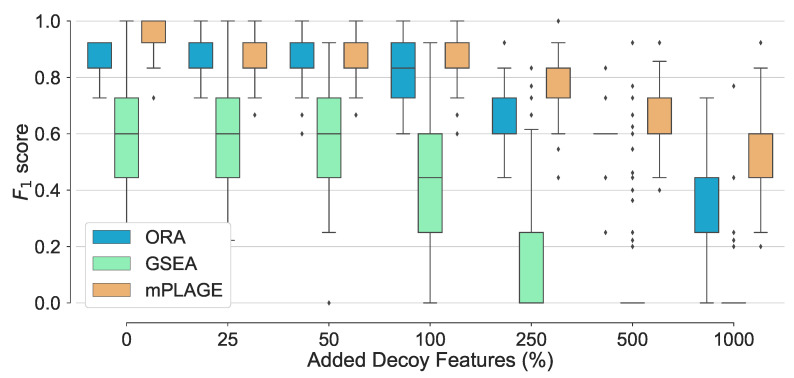
Overall performance of the different pathway ranking methods. Distribution of the $F_{1}$ for each method under increasing levels of decoy features is shown with outliers displayed as small diamonds. The performance of mPLAGE and ORA are roughly similar using 0–100% decoy levels, but mPLAGE performs better returning greater $F_{1}$-scores at higher decoy levels.

For all pathways across all levels of added decoy features, mPLAGE returns lower *p*-values compared to ORA and GSEA, which means that more significantly changing metabolite sets can be detected. This is similar to findings reported by Evangelou et al. [19] that compare methods for competitive tests for pathway analysis of gene sets and found that the power of all tested methods increased with the size of the pathway. Finally, $F_{1}$ score performance was evaluated. This summarises overall retrieval ability, taking into account the number of true positives, false positives, and false negatives of the methods being tested. Using this metric, it could be seen that the performance of ORA and mPLAGE were roughly similar using 0–100% levels of non-changing features (Figure 3). At higher levels of added decoy features of 250%, 500%, and 1000%, the $F_{1}$ scores of mPLAGE were consistently higher than ORA and GSEA. These results showed that a greater number of true positive pathways could be identified with mPLAGE than with ORA or GSEA. GSEA performed worst among the three methods tested, particularly showing its sensitivity to nonchanging or decoy features.

#### 2.1.4. Synthetic Experiment Results—Increasing Missing Features

**Figure 4:**
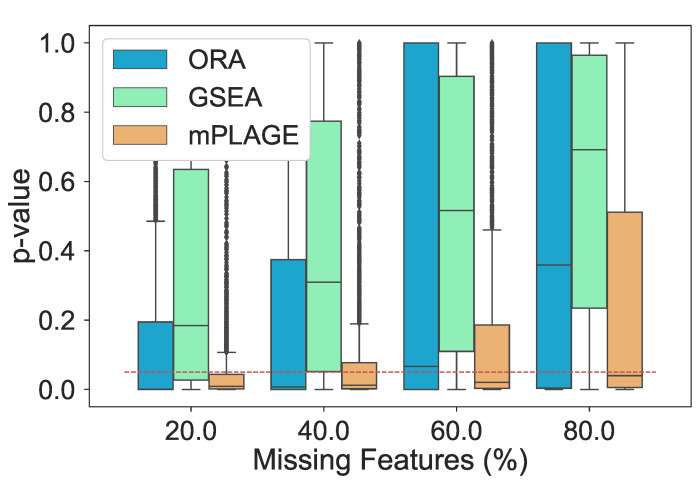
Resulting *p*-values from removing features from significantly changing pathways. The percentage of features randomly removed from the pathways increases from 20% to 80% and ORA, GSEA, and mPLAGE are compared. The red dashed line indicates a *p*-value threshold of 0.05. The results show that mPLAGE performs better, returning lower *p*-values compared to the alternatives, even in the presence of a large number of missing features.

**Figure 5:**
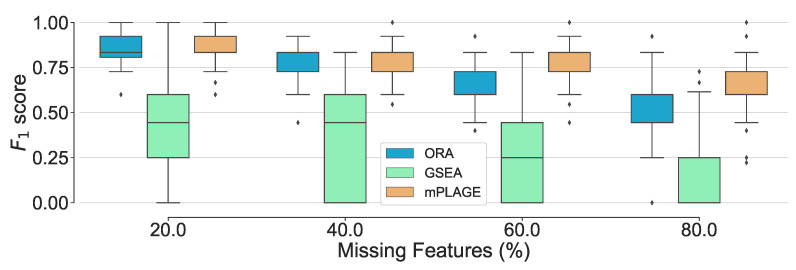
Overall performance of the different pathway ranking methods with missing features. Distribution of the $F_{1}$ scores for each method under an increasing proportion of missing features (20% to 80%) is shown. The results show that mPLAGE performs better, returning greater $F_{1}$-scores at higher proportions of missing features.

Additionally, the effect of introducing an increasing number of missing features from the pathways was explored. For this experiment, the level of decoy features was fixed to 100% in order to allow for an equal number of changing and nonchanging features. The percentage of features randomly missing from the data was indicated by *p*, and this value was varied from 20%, 40%, 60%, and 80% of the full data set. The different pathway ranking methods were run 500 times for each data set with each having a different value of *p*. The results in Figure 4 show that as the number of missing features increases, mPLAGE consistently returns lower mean *p*-values with smaller variances than ORA or GSEA. This suggests that mPLAGE is generally more robust to missing features than the alternative methods tested. These results were also supported by the $F_{1}$ score performance (Figure 5), where mPLAGE generally performed best even when large numbers of features were missing from the data.

### 2.2. Real Data Experiments

To evaluate the performance of the proposed method on actual complex biological data, PALS was used to analyse metabolomics data obtained from a study on human African trypanosomiasis (HAT) introduced in [20]. The causative agent of HAT is the parasite *Trypanosoma brucei*, which is transmitted to a human/mammalian host by the bite of the tsetse fly. Two data sets of samples collected from human blood plasma and cerebrospinal fluid (CSF) are available from the study, denoted as the Plasma and CSF (cerebrospinal fluid) data sets, respectively. The control group consists of parasite-free patients, while the two case groups are those with stage 1 (S1, parasites present in blood/lymphatics) or stage 2 (S2, parasites found in the CSF) trypanosomiasis.

#### 2.2.1. Case Study

Using the HAT data with PALS allows for complex clinical data to be explored and demonstrates how PALS can be used to draw relevant biological conclusions. The Plasma and CSF data sets from the HAT study, comprising of 20/17 control (C) samples and 20 samples from both S1 and S2, respectively, were uploaded and processed through the Polyomics Integrated Metabolomics pipeline (PiMP) [21]. PALS was easily integrated into PiMP and set to run automatically at the end of a data analysis workflow. PALS results from the HAT data revealed that a greater number of significantly changing pathways (*p*-value < 0.05) were found between the disease stages for the CSF than Plasma data (52/74, 42/128, and 42/143 for Plasma/CSF when comparing S1:C, S2:C, and S1:S2, respectively). In all of the comparisons, CSF samples have a greater number of changing pathways than those seen in the Plasma data. In particular, there is a notable difference in the changing pathways between the S1:C and S2:C/S1:S2 in the CSF. This can be explained by the fact that the parasites are only present in the CSF in S2 parasitemia and consequently more metabolic changes are expected in the CSF from S2 patients.

Looking at metabolic differences between S1 and S2 samples, the top-ranking PALS pathways in the CSF were heavily biased towards amino-acid metabolism (Supplementary Table S5). Changes in these pathways make sense for two reasons: firstly, because all parasites scavenge nutrients from their hosts and *T. brucei* is predicted to be auxotrophic for the amino acids arginine, glycine, histidine, isoleucine, leucine, lysine, phenylalanine, tryptophan, tyrosine, and valine [22], and secondly because it is unlikely that parasites will be present in the CSF of patients with a S1 infection. To support this finding, individual amino acids identified in the experiment were examined, and it was revealed that all of the amino acids required by *T. brucei* (listed above) (excluding glycine, which was undetected) were found to be decreasing in the CSF of patients with S2 of the disease (Supplementary Table S6). Interestingly, running the Plasma data through PALS did not show any bias towards amino acid metabolism, suggesting that the parasites potentially have a much more marked effect on the amino acid biosynthesis and availability on their host when it reaches the CSF.

It is known that when *T. brucei* reside in the human bloodstream, they are exposed to high levels of glucose, which they rely on for their energy metabolism [23]. This reliance on glucose changes when the parasites are resident in the insect midgut, as they have a less readily available supply of glucose, and instead switch to amino acids as their main carbon source [24]. Little is known about the metabolism of the parasites in the CSF, so it may be possible that amino acid usage is required to supplement the lower levels of glucose in the CSF (4.5 mM and 3 mM in Plasma and CSF, respectively [25]). Another reason that uptake of amino acids is evident in the CSF samples but not in the Plasma samples could be a result of amino acid concentrations being much lower in the CSF (4–50-fold lower for the nine amino acids than *T. brucei* are auxotrophic for [25]), and consequent salvaging of the nutrients by the parasites is more noticeable in the CSF.

#### 2.2.2. Real Data Experimental Setup

The HAT case study results demonstrate how the mPLAGE method in PALS identified significantly changing pathways that can be explained and interpreted as biologically relevant. In this section, the robustness of the different pathway ranking methods on this data is assessed. In a manner similar to the synthetic experiment, a range of missing feature proportions *p* was set from 20% to 80%.

After processing in PiMP, the complete Plasma and CSF data produced 15,584 and 8154 features, of which 1647 and 1132 had associated metabolite annotations, respectively. Therefore, *p* times the total number of features were randomly removed, and the rest were used as input for pathway analysis. Assuming that a particular pathway ranking $P_{m}$ returned by a method *m* on the complete data will be better than the ones obtained from the reduced data (with missing features), results from the complete data could be used as the true answers for evaluation. Here, true answers *T* are restricted to be the set of significant pathways above a threshold of 0.05 for their mPLAGE and ORA *p*-values, and a larger threshold of 0.25 for GSEA (following the suggestion in [6]).

Given *T* and $P_{m}$, performance in terms of precision, recall, and $F_{1}$ score can be computed as described in Section 2.1.2. This evaluation procedure was repeated 500 times. Comparisons were performed between the Stage1/Control groups on the Plasma data and between the Stage2/Control groups on the CSF data, where more changing pathways are expected to be found in the data set between the case and control groups as the disease progresses. The results are reported in Section 2.2.3.

#### 2.2.3. Robustness on Real Data

**Figure 6:**
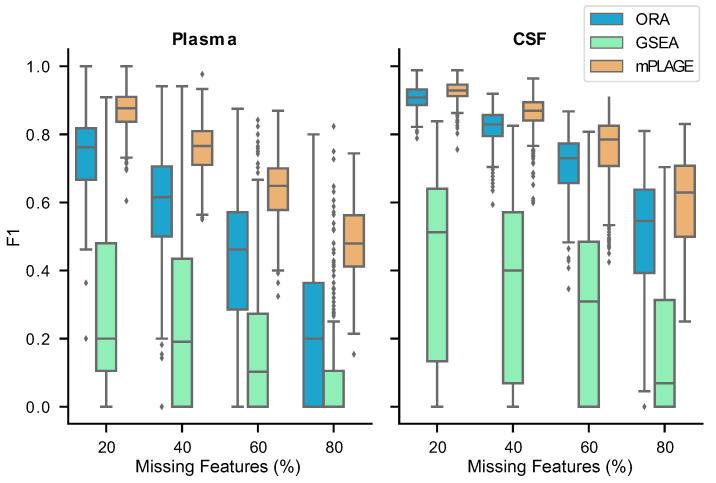
$F_{1}$ score results from the plasma and cerebrospinal fluid (CSF) samples from the human African trypanosomiasis (HAT) data set for varying percentages of missing features (proportion of full data set). A higher $F_{1}$ score means that the method is able to recover more of the original set of significant pathways correctly, even in the presence of missing features. The results show that mPLAGE performs better than the alternatives on real data.

For all methods, it can be noted that the $F_{1}$ score decreases as the proportion of missing features increase (Figure 6). This suggests that it becomes harder for all tested methods to reconstruct the original pathway ranking results obtained from the full data when fewer input features are available. On both the Plasma and CSF data, the pathway decomposition in mPLAGE performs best, demonstrating the most robust tolerance to missing features, followed by ORA. In both data sets, GSEA performs worst as a result of its sensitivity to noise. The results obtained for GSEA on the HAT data agree with those obtained for the synthetic data experiment.

At the highest proportion of missing features (80%) with the Plasma data, mPLAGE achieves a mean $F_{1}$ score of 0.48, while ORA and GSEA obtain scores of 0.20 and 0.08, respectively (Supplementary Table S7). Similarly, on the CSF data, mPLAGE achieves an $F_{1}$ score of 0.60 while ORA and GSEA obtain 0.50 and 0.16, respectively. ORA, on average, returns higher mean precision values among the benchmarked methods, but the better $F_{1}$ score of mPLAGE can be attributed to its generally superior recall performance while still offering competitive precision in comparison to ORA. The results here suggest that even when there are many missing features, mPLAGE is able to recover more original pathways in the full data using a small fraction of the features present in the original peak data, returning a higher number of true positives and fewer false positives and false negatives.

### 2.3. Analysis of Metabolite Sets: Molecular Families and Mass2Motifs

To demonstrate the analysis of other types of metabolite sets, PALS was used to assess the activity levels of potentially unknown metabolites grouped into molecular families (MFs) through their fragmentation spectral similarities, and into Mass2Motifs based on shared fragment and neutral loss features.

For the MF analysis, Global Natural Products Social Molecular Networking (GNPS) example data were taken from the American Gut Project [26]. From this, a subset of data was chosen from volunteers who had consumed different amounts of plant-based food. In this study, 35 significantly changing MFs (*p*-value ≤ 0.05) containing 10 or more molecules were found between the case (eating >30 plant-based foods a week) and control (<10 plant-based foods a week) groups. Of particular interest is one significantly changing MF (*p*-value ≤ 0.001) that was identified by PALS, consisting of 23 molecules that have steroid-related GNPS library hits. The intensities of the associated MS peaks in this steroid-related MF were found to be higher in the control group (for more details, see Supplementary Section S8), which could be a result of the abundance of steroids in meat, fish, and eggs in their diet. Most other significant MFs did not contain GNPS library hits but could represent potentially novel chemical classes.

To anaylse Mass2Motifs, a data set from the GNPS-MS2LDA workflow, consisting of 70 Rhamnacea species from two clades and various genera, was used [4]. In the original study, 25 Mass2Motifs were manually characterized and their distribution over the Rhamnaceae clades examined. The statistical significance of the results were easily obtained using PALS, whereas all previous efforts relied on manual interpretation.

Consistent with [4], PALS revealed that Mass2Motifs annotated with flavonoid-related substructures (i.e., rhamnocitrin, kaemfperol, flavonoid core framgent, and emodin) are all differently expressed between the *Rhamnus* and *Ziziphus* genera with features generally overrepresented in *Rhamnus* (for more details, see Supplementary Section S9). Similarly, the substructure set containing cyclopeptidic alkaloids was found to be overrepresented in *Ziziphus*. Differential metabolite sets for other genera could also be easily examined using PALS (for example, the Xylose or Arabinose moiety substructure was found to be differentially expressed between *Ventilago* and *Rhamnus*).

Finally, looking at Mass2Motifs in the American Gut Project data, 11 significant Mass2Motifs were prioritised using PALS, with one DE Mass2Motif being more abundant in “plant-eaters” and annotated as related to ferulic acid (12 members)—a molecule that is typically found in plants and could be linked to plant-based foods in this data set (see Supplementary Section S8 for more details).

## 3. Materials and Methods

### 3.1. Preparing Intensity Matrix

As input to activity level decomposition using mPLAGE (see Section 3.4), a peak intensity matrix *X* is constructed for each metabolite set, defined here as a collection of features that have been grouped in some manner, e.g., forming a particular metabolic pathway or molecular family. In *X*, each row represents a single feature and each column is a sample, grouped into user-defined factors/conditions. In such multisample LC–MS data sets, it is common to find missing data points across the samples. To address this, data imputation is performed on the intensity matrix as follows: if all of the samples in a single experimental factor have intensities of zero, these are replaced by a minimum intensity value (which can be set by the user), and if only some of the sample values in a factor are zero, then these are replaced by the mean value of the nonzero samples in that factor. As an option for data normalisation, the intensity matrix is subsequently transformed to log space and standardised using the preprocessing module in Scipy [27] such that each factor has a zero mean and unit variance across the samples. This has been shown to give good results in preserving the distribution of *p*-values of significantly changing metabolites [28].

### 3.2. Retrieving Pathway Data

**Figure 7:**
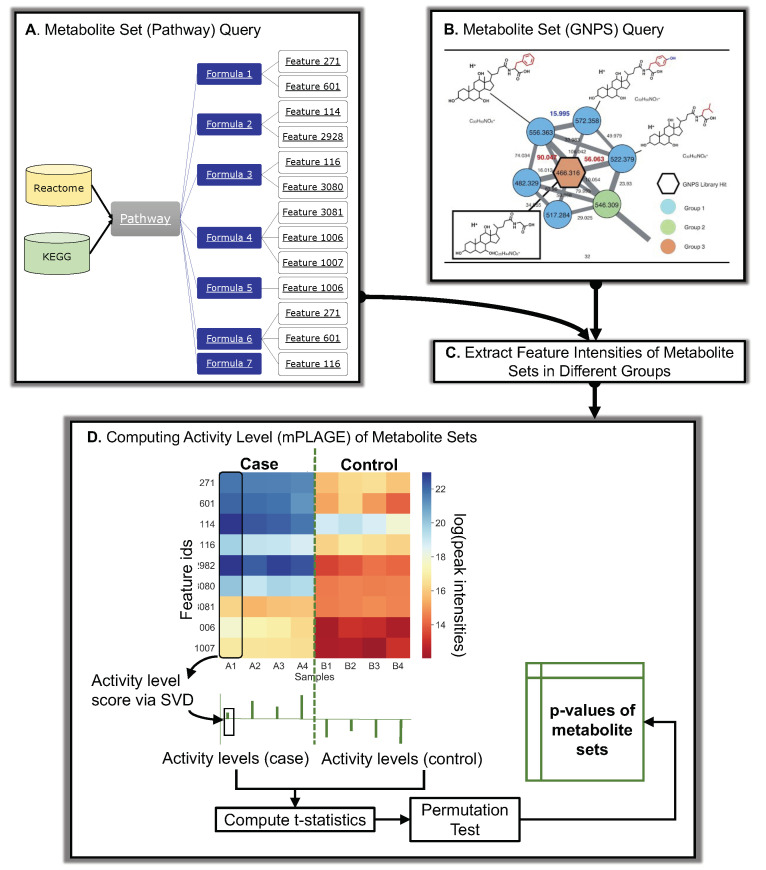
Overall schematic of Pathway Activity Level Scoring (PALS) using pathways and molecular families as an example of metabolite sets. (**A**) Database queries of pathways. Different databases (Reactome and KEGG) can be used to query pathways in PALS. Features are mapped to pathways through their formula annotations. For a set of compounds, all features sharing the same formula annotations of those compounds are used to construct the input intensity matrix for mPLAGE. (**B**) GNPS queries of molecular families or Mass2Motifs, potentially representing unknown chemical classes or substructures. (**C**) The intensities of features are extracted from peak data and assigned to experimental groups (**D**) The feature intensity matrix and mPLAGE method for a metabolite set. In the matrix, feature intensity levels are shown as a heat map ranging from blue (high) to red (low) peak intensities. The feature intensity matrix is decomposed via SVD to obtain the activity level (AL) scores from a set of metabolites in a sample. The collective AL scores are used to calculate the subsequent mPLAGE *p*-values between the different factor groups. The dashed green line is used to split samples grouped as different factors.

Metabolites, pathways, and the mapping of metabolites to pathways are obtained in PALS by querying from a local copy of the KEGG database [29] or by querying a Reactome instance running on top of a local Neo4j graph database (Figure 7A). For Reactome, queries are created using Cypher, the query language used in the Neo4j graph database that hosts the Reactome data, to retrieve pathway information. Reactome is regularly updated, with the entire database available from the Reactome website https://reactome.org/ (accessed on 5 February 2021) [30].

The presence of isomeric and isobaric compounds (same mass, different structure/ molecular formula, respectively) in a sample means that multiple features with close m/z values can be annotated with the same molecular formula, although their associated peaks are actually produced by different compounds in the MS. Resolving the actual molecular identities of annotated features is a challenging problem, even at a high resolution [9]. To ensure coverage, PALS represents compounds using their chemical formulae when mapping features to pathways. Features are annotated as compound formulae and these are linked to the metabolites in the metabolite set/pathway of interest. To minimise false positive identifications of compounds from features, only those annotated as the most commonly observed adducts were selected: protonated (M+H)${}^{+}$ and deprotonated (M-H)${}^{-}$ from positive and negative ionisation modes, respectively.

### 3.3. Retrieving Molecular Family and Mass2Motif Data

The decomposition approach employed by mPLAGE is not limited to the analysis of pathways. In fact, any user-defined grouping of features, where each metabolite set can be represented as the intensity matrix *X* (features vs. samples), can be used for for activity level decomposition. Here, two other approaches for producing metabolite sets by grouping features in a data-dependent fashion, in contrast to pathways that rely on prior knowledge, are described. The first method uses molecular networking (FBMN, [31]) to cluster peak fragmentation spectra by their similarities and produce the groups of peaks known as molecular families (MF). The second method employs a topic-modelling approach (MS2LDA, [5]) to generate groups of peaks (called Mass2Motifs) based on the shared presence of fragment and neutral loss features in their fragmentation spectra. Both FBMN and MS2LDA are accessible as workflows from Global Natural Products Social Molecular Networking (GNPS, [2]), which provides community resources to run large-scale molecular networking and annotations of spectra. To analyse both MFs and Mass2Motifs, Python code is provided in PALS to retrieve information on the metabolite sets as well as the peak intensity table from GNPS (Figure 7B). This is then used to extract features and construct the input matrices for mPLAGE analysis (Figure 7C).

### 3.4. Decomposing Metabolite Set Activity Levels Using mPLAGE

The initial activity level (AL) score for a set of metabolites is computed using singular value decomposition (SVD) following the method described for PLAGE [7], and adapted for metabolomics data (mPLAGE), as described. Similar to the way that gene expression data can be decomposed into metagenes and activity levels in PLAGE [7], the intensity matrix *X* can be used to determine an AL score for a compound in a pathway or set of metabolites in mPLAGE. This is calculated as the level of the first metacompound from the SVD of the peak intensity matrix (Figure 7D) as follows. Given an intensity matrix *X* for a group of metabolites (where rows are the features annotated with formulae and columns are the samples in the data set), the decomposition of *X* can be written as X=UΣV where columns in *U* are the orthonormal left singular vectors representing “metacompounds’’, Σ contains the diagonal matrix of singular values arranged in descending order, and rows in *V* are the right singular vectors representing the contribution of the corresponding metacompounds to each sample. The AL score in a sample *j* is given by $v_{j}$, where *v* is the first row in *V*, which is a vector of coefficients representing the activity levels of the first metacompound in *U* (having the largest singular value) across samples. The singular values σ in Σ scale the metacompounds so that the original data *X* can be constructed—in fact, the square of each $\sigma_{i}$ is a measure of the variance accounted for by each metacompound *i*. For more details, see [7].

To perform activity level analysis, two experimental factors are compared—for example, a control versus a condition. This is achieved by using the AL scores obtained from the decomposition step to calculate the *t*-statistic for each metabolite set across the varying experimental factors (Figure 7D). As in [7], random permutations of the sample labels are performed to obtain the null distributions of *t*-statistics. However, it is observed that the tail end of the distribution of permuted *t*-statistics occasionally contains some extreme values that could skew results. As part of mPLAGE, an enhancement to the original PLAGE approach is introduced which produces better calibrated *p*-values. The minimum and maximum *t*-statistics from each permutation test are modelled using the generalised extreme value (GEV) distribution [32], with parameter fitting using maximum-likelihood estimation. The *t*-statistic of a pathway or set of metabolites is then evaluated against the fitted density in order to produce the final mPLAGE *p*-values of the metabolite sets.

### 3.5. Software Implementation

To facilitate the use of the activity analysis described in this project, PALS (Pathway Activity Level Scoring) software was developed. PALS can be run either as a standalone tool, imported as a Python library, or used via a Web interface allowing users with a wide range of expertise and requirements to use it (input format detailed in Supplementary Section S1). To assist beginners, a Web interface is provided to run PALS in an intuitive and user-friendly manner (refer to Supplementary Section S2 for more details). Intermediate users can run PALS as a standalone command-line tool and incorporate PALS into a workflow as part of a custom script. In this mode, feature and annotation CSV files are input on the command line along with experimental design parameters, and a variety of other available parameters, accessible through command-line options (detailed in Supplementary Section S2). Pathway ranking results are output as a CSV file listing pathways, or metabolite sets, and their *p*-values. Expert users can also incorporate the PALS library directly in their Python application or use it in an interactive data analysis environment such as Jupyter Notebook [33]. A comprehensive tutorial describing how to use PALS as a library is available from the project Web site.

## 4. Conclusions

This work describes PALS, a comprehensive system that can be used to prioritise metabolite sets, that is freely available at https://pals.glasgowcompbio.org/ (accessed on 5 February 2021). From PALS, users obtain the ranking of changing pathways, or user-defined sets of metabolites, between experimental factors. Database queries that map compounds to pathways are integrated into the system using either KEGG or Reactome databases, while code is also available to automatically retrieve measurement data and metabolite sets of molecular families and Mass2Motifs from GNPS and perform activity measurements. Manuscripts comparing pathway ranking methods for metabolomics are still relatively rare. One such manuscript [34] compares the performance of overrepresentation analysis (ORA) tools, revealing that tools using ORA for analysis give consistently similar results while incorrect versions of the databases can affect the results more. With PALS, a systematic comparison using synthetic and real data was performed for the mPLAGE method against two different and widely used methods for pathway ranking in metabolomics ORA and GSEA. Comparing these different methods show that mPLAGE is consistently more robust in the presence of noisy and missing features, a common problem in LC–MS peak data. In addition, by choosing to use PALS with the Reactome DB, users can consistently access the most recent version of the DB, avoiding any version issues.

It is also worth emphasising that unlike many tools surveyed here that specifically deal with pathways, PALS could be easily used to prioritise metabolite sets derived from many sources, whether based on knowledge (pathways) or driven by fragmentation data (molecular families and Mass2Motifs). To our knowledge, this is a unique capability not present in other tools. PALS also presents a comprehensive Python-based library that can be easily run in several modes: as a standalone tool, an interactive Web application (PALS Viewer), or a Python library. This allows PALS to be embedded in many different contexts: as part of a larger Web application, or a custom Python-based workflow and scripts, or to be imported directly into interactive computing environments like Jupyter. As a demonstration of its utility and versatility, the PALS library has been integrated and actively used in several projects such as PiMP [21], FlyMet (flymet.org (accessed on 5 February 2021) [35]), and WebOmics (webomics.glasgowcompbio.org (accessed on 5 February 2021) [36]). To support these projects, PALS was extended to analyse not just metabolite sets, but also gene sets as well as protein sets from Reactome, since the mPLAGE approach used for activity level decomposition could easily generalise to other types of omics data too.

## Data Availability

The data referenced in Section 2.2 is available at https://www.ebi.ac.uk/metabolights/MTBLS413 (accessed on 5 February 2021).

## Supplementary Materials

A variety of additional text, tables and figures are available online at https://www.mdpi.com/2218-1989/11/2/103/s1. Supplementary Section S1: File format and data imputation, Supplementary Section S2: Running PALS, Supplementary Section S3: Synthetic data generation, Supplementary Section S4: Benchmark methods, Supplementary Table S5: Top 30 ranking pathways from the HAT CSF dataset, Supplementary Table S6: Metabolites annotated in the KEGG aminoacyl-tRNA biosynthesis pathway, Supplementary Table S7: Precision and recall on real HAT data, Supplementary Section S8: Analysis of Differentially Expressed Molecular Families and Mass2Motifs from the AGP Dataset, Supplementary Section S9: Analysis of Differentially Expressed Mass2Motifs from the Rhamnaceae Dataset.
