# Supplementary material for: Ranking Metabolite Sets by Their Activity Levels
Source: Metabolites. 2021 Feb 11;11(2):103. doi: 10.3390/metabo11020103 (PMC7916825; doi:10.3390/metabo11020103)
Supplement: Supplementary file 1 [file metabolites-11-00103-s001.pdf]

Ranking metabolite sets by their activity levels

Karen McLuskey[a], Joe Wandy[a], Isabel Vincent[b], Justin J. J. van der Hooft[c], Simon Rogers[d], Karl Burgess[e], and Rónán Daly[a,*]

[a]Glasgow Polyomics, University of Glasgow, Glasgow, G61 1BD, United Kingdom
[b]IBioIC, Strathclyde Institute of Pharmacy and Biomedical Sciences, University of Strathclyde, Glasgow, G1 1XQ, United Kingdom.
[c]Bioinformatics Group, Department of Plant Sciences, Wageningen University, Wageningen, The Netherlands.
[d]School of Computing Science, University of Glasgow, Glasgow G12 8RZ, United Kingdom.
[e]Centre for Synthetic and Systems Biology, School of Biological Sciences, University of Edinburgh, United Kingdom.

November 26, 2020

Supplementary Section S1: File format and data imputation

To use PALS, users have to provide information on feature intensities, feature annotations and the experimental design. Feature intensities should be provided as a matrix where the first column contains the feature IDs and further columns represent individual samples. When uploading a CSV file to the PALS Viewer or via the command line, the second line of this file should be used to indicate which groups this sample belongs to.

For example, the intensity matrix takes the form of:

```
peak_id,A001C.mzXML,A001P.mzXML,A002P.mzXML,A003C.mzXML,A004C.mzXML,A005C.mzXML,
    A008C.mzXML,A008P.mzXML,A009C.mzXML,A010C.mzXML,A010P.mzXML,A011C.mzXML
group, Control,Stage_2,Stage_2,Control,Control,Control,
    Control,Stage_1,Control,Control,Stage_1,Control
36186,612715072,408723328,356592704,575874816,627733440,399707168,
    621499008,545524352,522808352,557363328,541595328,476640800,531225312,535235328
36187,272679552,222055984,187961552,254896960,274777440,204597328,
    271147648,244269440,246383280,262514720,255993888,228511584
```

In addition to the feature intensities, users also provide a list of compound annotations assigned to features (features that do not have annotations will not be used for pathway analysis). As a result of the uncertainty during identification, multiple features IDs could be mapped to multiple compound IDs and *vice versa*. As such, annotations are provided as another matrix having two columns. The first column (or DataFrame index) is the peak ID while the second column is the assigned metabolite annotation as either KEGG or ChEBI database IDs.

```
peak_id,entity_id
36883,C00111
37231,C00111
37309,C00661
37231,C19156
36368,C02718
37714,C05100
```

Supplementary Section S2: Running PALS

PALS can be run in a variety of ways: from the command-line, from the Web interface (PALS Viewer) as well as imported directly as a Python library. Users should begin by first installing PALS using the following command: *pip install pals-pathway*, This retrieves the latest stable version of PALS from the Python Package Index.

S2.1 Running PALS from the command-line

To run PALS from the command-line, the script *pals/run.py* is used. This script accepts a number of parameters, documented here (* indicates required parameters):

```
usage: run.py [-h] --db {PiMP_KEGG,COMPOUND,ChEBI,UniProt,ENSEMBL}
              --comparisons COMPARISONS [COMPARISONS ...]
              [--min_replace MIN_REPLACE]
              [--species {Arabidopsis thaliana,Bos taurus,Caenorhabditis elegans,
              Canis lupus familiaris,Danio rerio,Dictyostelium discoideum,
              Drosophila melanogaster,Gallus gallus,Homo sapiens,Mus musculus,
              Oryza sativa,Rattus norvegicus,Saccharomyces cerevisiae,Sus scrofa}]
              [--use_all_reactome_pathways] [--connect_to_reactome_server]
              {PLAGE,ORA,GSEA} intensity_csv annotation_csv output_file
```

method *
Pathway ranking method to use, e.g. PLAGE, ORA or GSEA.

intensity_csv *
Input intensity CSV file (see Supplementary Section S1).

annotation_csv *
Input annotation CSV file (see Supplementary Section S1).

output_file *
Output pathway ranking file.

–db *
The pathway database to use. Valid choices are as follows.

- *PiMP_KEGG*: KEGG compound database exported from PiMP.
- *COMPOUND*: Reactome compound database matching by KEGG ids.
- *ChEBI*: Reactome compound database matching by ChEBI ids.
- *UniProt*: Reactome protein database matching by UniProt ids.
- *ENSEMBL*: Reactome gene database matching by ENSEMBL ids.

Note that *PiMP_KEGG*, *COMPOUND* and *ChEBI* are for metabolomics use, while *UniProt* and *ENSEMBL* are for proteomics and transcriptomics use respectively and are not considered in this paper (refer to the project Web site for more information).

–comparisons *
Specifies the comparisons to make, e.g. *–comparisons Stage_1/Control Stage_2/Control* to specify Stage 1 (case) vs control, as well as Stage_2 (case) vs control.

–min_replace
: The minimum intensity value for data imputation, e.g. *–min_replace 5000*. Defaults to 5000.

–species
: Species name for Reactome pathway query, e.g. *–species "Homo sapiens"*. Defaults to Homo Sapiens.

–use_all_reactome_pathways
: Whether to use all pathways for Reactome pathway query. If this option is not used, only metabolic pathways will be queried.

–connect_to_reactome_server
: Whether to connect to an instance of Neo4j server hosting Reactome database (online mode). If not specified, then offline mode (using a downloaded copy of selected Reactome pathways) will be used.

S2.2 Running PALS Viewer

To assist in results interpretation, *PALS Viewer* provides a user-friendly Web-based interface to run PALS and analyse pathway ranking results as well as inspect significantly changing pathways. It can be installed and run locally or accessed from the project Web site, and can be used analyse three types of metabolite sets: pathways, molecular families (MFs) and Mass2Motifs. An online instance of PALS Viewer can also be accessed from the project Web site at `https://pals.glasgowcompbio.org/`.

For pathway analysis, users start by uploading their intensity and annotation CSV files; input format is detailed in Supplementary Section S1 but example files are also available directly from PALS Viewer. Users can then easily configure the appropriate parameters such as experimental design, preferred species and database. Once the pathway decomposition is run the analysis results are shown in an interactive pathway ranking table (Figure 1).

Each entry in the table shows pathways, their corresponding mPLAGE p-values and the number of formula hits. These can be used for sorting and filtering by chosen thresholds. Choosing to order the pathways by the p-values means small but consistent changes in a group of pathway metabolites appear near the top (most interesting), even if the changes in the individual metabolites are modest.

Selecting a pathway from the Pathway Browser reveals the Reactome (or KEGG) pathway diagram. Information on the fold changes of annotated compounds are submitted to Reactome Analysis Service and mapped onto the Reactome pathway diagram, which is linked from the Viewer. As an option, users can also display a heatmap of the annotated formula hits in the pathway. A heatmap displaying the feature intensity levels of the annotated formula hits, across all samples grouped by experimental factors, in the pathway is also shown. This allows the user to easily visualise the changes in the pathway metabolites between experimental factors.

To analyse both MFs and Mass2Motifs using PALS Viewer users must provide links to an existing GNPS FBMN result containing the MS1 peak table (for the feature intensity matrix) and a csv file containing information on the sample groups. For Mass2Motifs analysis, users provide an addition link to a GNPS MS2LDA result which describes the grouping of features into Mass2Motifs. As for pathway analysis, example files are given in PALS Viewer. This data

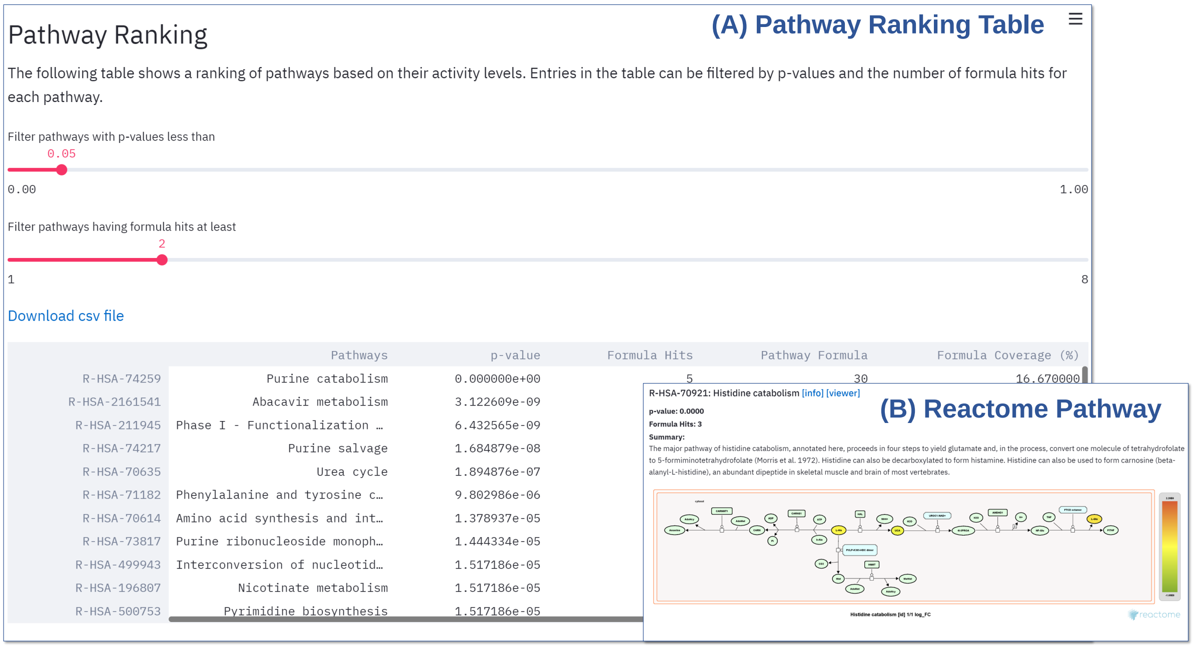

Figure 1: An example of PALS Viewer results for analysing pathways in the CSF HAT data is shown: **(A)** Activity results are shown in the Pathway Ranking table. Entries can be sorted and filtered by p-value threshold or the number of formula hits. **(B)** An example Reactome pathway selected from the Pathway Browser. Fold change values are mapped onto the pathway diagram using Reactome Analysis Service.

is loaded into PALS, and features are allocated to metabolite sets according to their groups (note that a feature can be assigned to only one MF but to multiple Mass2Motifs). Activity level decomposition is then performed on the metabolite sets using mPLAGE. The results are presented in PALS Viewer in a similar manner to pathways: MFs or Mass2Motifs are shown in a ranked interactive table next to their mPLAGE p-values. Upon selecting a metabolite set (either an MF or a Mass2Motif), a heatmap is displayed showing the intensity values of member features across samples, as well as any additional metadata retrieved from GNPS or MS2LDA.

S2.3 Using PALS as a library

PALS can be imported as a Python library and incorporated into your own Python application. This is illustrated in the following code snippet (for additional documentation and tutorials, please refer to the project Web site):

```
from pals.PLAGE import PLAGE
from pals.ORA import ORA
from pals.GSEA import GSEA
from pals.common import *
from pals.feature_extraction import DataSource

# TODO: correctly initialise the following data structures for your data
# See Section S2.3.1 below.
int_df = pd.DataFrame()
```

```
annotation_df = pd.DataFrame()
experimental_design = {}

# Using Reactome pathways matching by KEGG ID
database_name = 'COMPOUND'

# If true, we limit to metabolic pathways only. Otherwise all pathways will be queried.
reactome_metabolic_pathway_only = True

# If true, we use online mode that queries Reactome on a local Neo4j server.
# Otherwise offline mode will be used (using downloaded database files).
reactome_query = True

# Minimum intensity value for data imputation
min_replace = 5000

ds = DataSource(int_df, annotation_df, experimental_design, database_name,
                reactome_species=reactome_species,
                reactome_metabolic_pathway_only=reactome_metabolic_pathway_only,
                reactome_query=reactome_query, min_replace=min_replace)

# choose a method
method = PLAGE(ds)
# method = ORA(ds)
# method = GSEA(ds)

df = method.get_pathway_df()
```

S2.3.1. Data structures

When PALS is used programatically, pandas DataFrames storing the intensity and annotation data, along with a dictionary describing the experimental design, can be passed directly to the program.

In the example above, *int_ df* is the intensity DataFrame containing feature intensity information described in Section S1 (with the second line of grouping information omitted). Similarly *annot_ df* is the annotation DataFrame containing feature annotations as described in Section S1. The experimental design data in *experimental_ design* contains information on 'groups', which relates all samples in a particular experimental factor together as well as 'comparisons', which describes the desired comparisons for the PALS analysis in terms of a case and a control. An example of this can be found below:

```
experimental_design = {
    'comparisons': [
        {'case': 'Stage_1', 'control': 'Control', 'name':  'Stage_1/Control'},
        {'case': 'Stage_2', 'control': 'Control', 'name': 'Stage_2/Control'},
        {'case': 'Stage_2', 'control': 'Stage_1', 'name': 'Stage_2/Stage_1'}
    ],
    'groups': {
```

```
        'Stage_1': [
            'A008P.mzXML',
            'A009P.mzXML',
            'A010P.mzXML'
        ],
        'Stage_2': [
            'A001P.mzXML',
            'A002P.mzXML',
            'A003P.mzXML'
        ],
        'Control': [
            'A001C.mzXML',
            'A002C.mzXML',
            'A003C.mzXML'
        ],
    }
}
```

Supplementary Section S3: Synthetic data generation

Synthetic pathway data is constructed to have differentially expressed intensity matrix (an example is shown in the figure below). Two groups (case and control) are included. The log intensity values of the control group is drawn from a normal (Gaussian) distribution with mean 20.0 and standard deviation 5.0, while for the case group, a normal distribution with mean 40.0 and standard deviation 5.0 is used. Each pathway is associated with the specified number of metabolites within the set: $2, 4, 6, 10, 20, 40, 80$. To simplify the problem of assigning features to metabolites, we assume a one-to-one correspondence between a feature and a metabolite (one metabolite produces exactly one feature). A synthetic pathway is labelled by the number of metabolites assigned to it (e.g. pathway *twenty* has 20 metabolites and therefore 20 features). In addition, 100 background pathways containing only noise (showing no significant changes between the case and control groups) were generated. The number of metabolites in a background pathway was randomly drawn with a uniform probability from 5 to 50, while the log intensity value in the random pathway is drawn from a normal distribution with mean 0 and standard deviation of 1. To simulate missing features, which often occurs in real data due to improper parameters used in peak picking or other preprocessing steps in the pipeline (e.g. setting an intensity filter threshold that is too low), features are also randomly removed from pathways with a uniform probability of 0.2. The total number of pathways evaluated in the synthetic data experiment is 107, composed of seven significantly changing pathways and 100 noisy pathways.

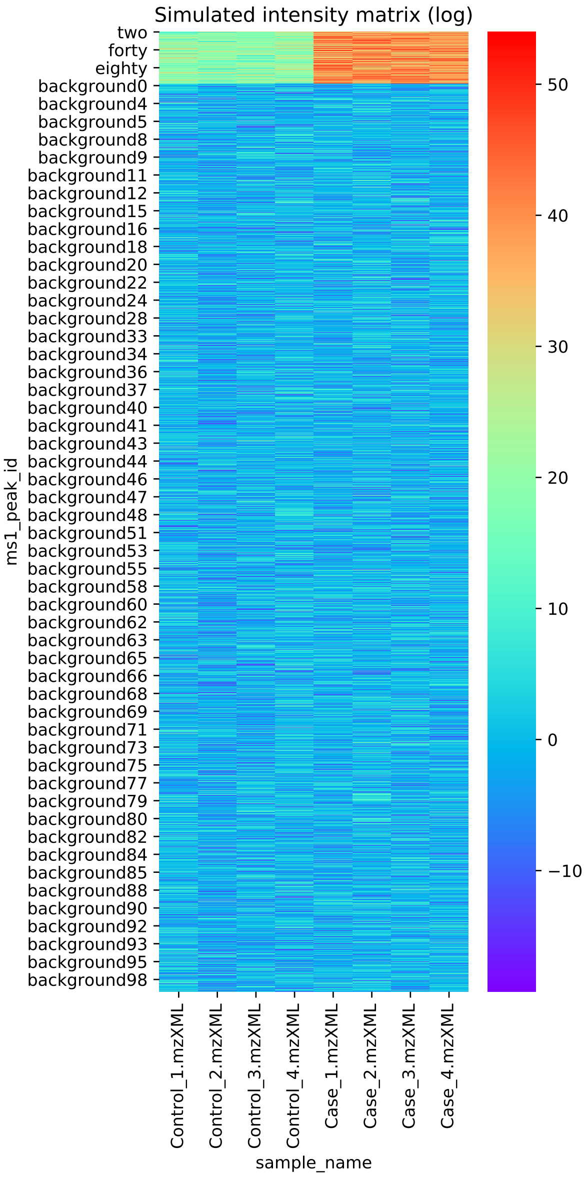
Simulated intensity matrix (log)
ms1_peak_id
two
forty
eighty
background0
background4
background5
background8
background9
background11
background12
background15
background16
background18
background20
background22
background24
background28
background33
background34
background36
background37
background40
background41
background43
background44
background46
background47
background48
background51
background53
background55
background58
background60
background62
background63
background65
background66
background68
background69
background71
background73
background75
background77
background79
background80
background82
background84
background85
background88
background90
background92
background93
background95
background98
Control_1.mzXML
Control_2.mzXML
Control_3.mzXML
Control_4.mzXML
Case_1.mzXML
Case_2.mzXML
Case_3.mzXML
Case_4.mzXML
sample_name
50
40
30
20
10
0
−10

Supplementary Section S4: Benchmark methods

The ORA method used for benchmarking is briefly summarised here: for each pathway, the number of significantly changing metabolites (above a p-value threshold of 0.05) is counted. Hypergeometric test is used to assess the probability of over-representation of significantly changing metabolites in that pathway. This procedure is repeated for all pathways, and the Benjamini-Hochberg correction is used to correct for multiple t-tests in the final results. For more details, refer to [2].

For GSEA, the GSEApy python package (`https://github.com/zqfang/GSEApy`), which implements the Gene-Set Enrichment Analysis algorithm in [5], was used. The steps in GSEA include the calculation of an enrichment score (ES). This is achieved by ranking metabolites according to the correlation of their feature intensity profiles to different experimental factors. Subsequently, a permutation test is performed to estimate the significance of the observed ES to the null hypothesis by randomly permuting factor labels, and correcting for multiple hypothesis testing by computing the false discovery rate. Following the original GSEA paper [5], the recommended number of 1000 permutations was used, as well as permuting the phenotype (sample) labels rather than the gene labels during permutation test. To produce the initial ranking of metabolites, we use the signal-to-noise ratio, which is also used by default in GSEA.

Supplementary Table S5: Top 30 ranking pathways from the HAT CSF dataset

Pathway Name	AF	TPF	FC %	S1/S2	S2/C	S1/C
Pantothenate and CoA biosynthesis	11	25	44	0.0E+00	6.6E-26	9.7E-01
Cyanoamino acid metabolism	13	40	32.5	0.0E+00	2.8E-11	4.1E-02
Arginine and proline metabolism	23	79	29.11	0.0E+00	1.3E-09	6.8E-04
Purine metabolism	9	78	11.54	0.0E+00	2.4E-09	4.3E-01
Alcoholism	3	10	30	0.0E+00	2.6E-09	9.3E-02
Aminoacyl-tRNA biosynthesis	14	23	60.87	0.0E+00	5.5E-09	1.2E-03
Cocaine addiction	2	7	28.57	0.0E+00	5.8E-09	2.5E-01
Amphetamine addiction	2	9	22.22	0.0E+00	7.8E-09	2.6E-01
Amyotrophic lateral sclerosis (ALS)	2	10	20	0.0E+00	2.1E-08	2.6E-01
Protein digestion and absorption	14	42	33.33	0.0E+00	3.3E-08	3.1E-03
Mineral absorption	10	26	38.46	0.0E+00	2.0E-07	4.2E-02
ABC transporters	18	80	22.5	0.0E+00	2.3E-07	1.7E-02
Anticonvulsants	1	4	25	0.0E+00	2.8E-07	8.1E-01
Alanine, aspartate and glutamate metabolism	7	23	30.43	0.0E+00	4.1E-07	4.2E-01
African trypanosomiasis	1	7	14.29	0.0E+00	4.4E-07	8.3E-01
Novobiocin biosynthesis	2	25	8	0.0E+00	9.3E-07	7.5E-01
Cysteine and methionine metabolism	7	52	13.46	0.0E+00	1.3E-06	7.4E-01
Neuroactive ligand-receptor interaction	4	50	8	0.0E+00	1.5E-06	9.8E-01
Indole alkaloid biosynthesis	1	30	3.33	0.0E+00	2.0E-06	9.0E-01
Histidine metabolism	7	41	17.07	0.0E+00	5.2E-06	1.0E-03
Phenylalanine, tyrosine and tryptophan biosynthesis	9	30	30	0.0E+00	1.1E-05	5.4E-03
Phenylalanine metabolism	12	55	21.82	0.0E+00	1.9E-05	3.6E-02
beta-Alanine metabolism	7	31	22.58	0.0E+00	2.9E-05	2.4E-02
Ubiquinone and other terpenoid-quinone biosynthesis	6	56	10.71	0.0E+00	1.5E-04	1.1E-04
Glutathione metabolism	5	29	17.24	0.0E+00	4.3E-04	3.9E-02
Caprolactam degradation	7	19	36.84	2.5E-25	8.2E-01	1.6E-10
Glycine, serine and threonine metabolism	9	41	21.95	1.6E-22	8.4E-07	3.6E-01
Bacterial chemotaxis	1	5	20	1.5E-21	1.5E-05	9.9E-01
Sphingolipid metabolism	1	10	10	4.4E-21	2.4E-05	9.9E-01
Glyoxylate and dicarboxylate metabolism	8	48	16.67	4.8E-21	9.1E-06	7.1E-02

The top 30 best ranking pathways based on the PALS of the stage 1 (S1) compared to stage 2 (S2) in the cerebrospinal fluid (CSF) of patients with Human African Trypanosomiasis (HAT). The annotated formula (AF) found in the dataset and belonging to a particular pathway is shown along with the total formula expected in a pathway (TPF) and the percentage of the formula coverage (FC %). In the analysis, comparisons were made for between S1 and S2 along with S2 and S1 compared to the control (C) samples. The total number of KEGG pathways returned for this experiment was 162 and from these many of those involved in amino-acid metabolism were found to be highly significant.

Supplementary Table S6: Metabolites annotated in the KEGG aminoacyl-tRNA biosynthesis pathway

**Amino acid**	**Stage2/Stage1 (intensity)**
L-Arginine	Significant decrease
L-Asparagine	Significant increase
L-Aspartate	Insignificant decrease
L-Glutamate	Significant increase
L-Glutamine	Insignificant decrease
L-Histidine	Significant decrease
L-Isoleucine	Insignificant decrease
L-Leucine	Significant decrease
L-Lysine	Significant decrease
L-Methionine	Insignificant decrease
L-Phenylalanine	Significant decrease
L-Proline	Significant increase
L-Serine	Significant decrease
L-Threonine	Significant decrease
L-Tryptophan	Significant decrease
L-Tyrosine	Significant decrease
L-Valine	Insignificant decrease

The metabolites annotated in the KEGG aminoacyl-tRNA biosynthesis pathway in the CSF of HAT patients. Some metabolites show a significant increase or decrease, while others show an insignificant decrease in intensities between stage 1 and stage 2. All of the metabolite features were identified using in-house standards apart from L-Tyrosine that was identified through fragmentation and L-Aspartate for which no identification (only annotation was) was obtained.

Supplementary Table S7: Precision and recall on real HAT data

Data	Missing Features	Method	Mean Prec.	Mean Recall	Mean F_1
Plasma	0.2	ORA	0.83	0.71	0.74
		GSEA	0.71	0.22	0.30
		PALS	**0.86**	**0.88**	**0.87**
	0.4	ORA	**0.77**	0.53	0.59
		GSEA	0.55	0.18	0.24
		PALS	**0.77**	**0.76**	**0.76**
	0.6	ORA	**0.77**	0.32	0.42
		GSEA	0.43	0.12	0.16
		PALS	0.69	**0.61**	**0.64**
	0.8	ORA	0.53	0.14	0.20
		GSEA	0.20	0.06	0.08
		PALS	**0.61**	**0.42**	**0.48**
CSF	0.2	ORA	**0.95**	0.87	0.91
		GSEA	0.52	0.46	0.41
		PALS	0.94	**0.91**	**0.93**
	0.4	ORA	**0.92**	0.75	0.82
		GSEA	0.52	0.37	0.34
		PALS	0.91	**0.83**	**0.86**
	0.6	ORA	**0.90**	0.60	0.71
		GSEA	0.41	0.32	0.29
		PALS	0.87	**0.69**	**0.76**
	0.8	ORA	**0.87**	0.38	0.50
		GSEA	0.27	0.17	0.16
		PALS	0.81	**0.49**	**0.60**

Mean precision, recall and F_1 score for the different methods under various missing features proportion on the Plasma and CSF data. The highest values (and ties) for precision, recall and F_1 score for each experimental setting is highlighted in bold.

Supplementary Section S8: Analysis of Differentially Expressed Molecular Families and Mass2Motifs from the AGP Dataset

PALS was run on the following GNPS-FMBN [4] results from a previous analysis of a subset from the American Gut Project (AGP) dataset [3] comparing volunteers who eat differential amounts of plant-based food. The case group was selected to be those who eat more than 30 plant-based foods a week, while the control consists of those eating less than 10 plant-based foods a week.

For analysis, the GNPS-FBMN data available from `https://gnps.ucsd.edu/ProteoSAFe/status.jsp?task=0a8432b5891a48d7ad8459ba4a89969f` was used to extract MS1 peak table and grouping information. Sample metadata CSV is provided at `https://github.com/glasgowcompbio/PALS/raw/master/notebooks/test_data/AGP/AG_Plants_extremes_metadata_df.csv`. Analysis was performed using PALS Viewer at `https://pals.glasgowcompbio.org/app`. The following Jupyter notebook can also be used to perform the same analysis: `https://github.com/glasgowcompbio/PALS/blob/master/notebooks/GNPS_analysis.ipynb`.

In total, 35 significantly changing MFs containing 10 or more molecules were found to be DE between case and control groups. We found a notable Molecular Family containing steroid-related molecules of interest that is statistically significant (p-value $\leq$ 0.001). This is plotted below, with the corresponding GNPS cluster id labelled green in the plot axes and also listed in the following table.

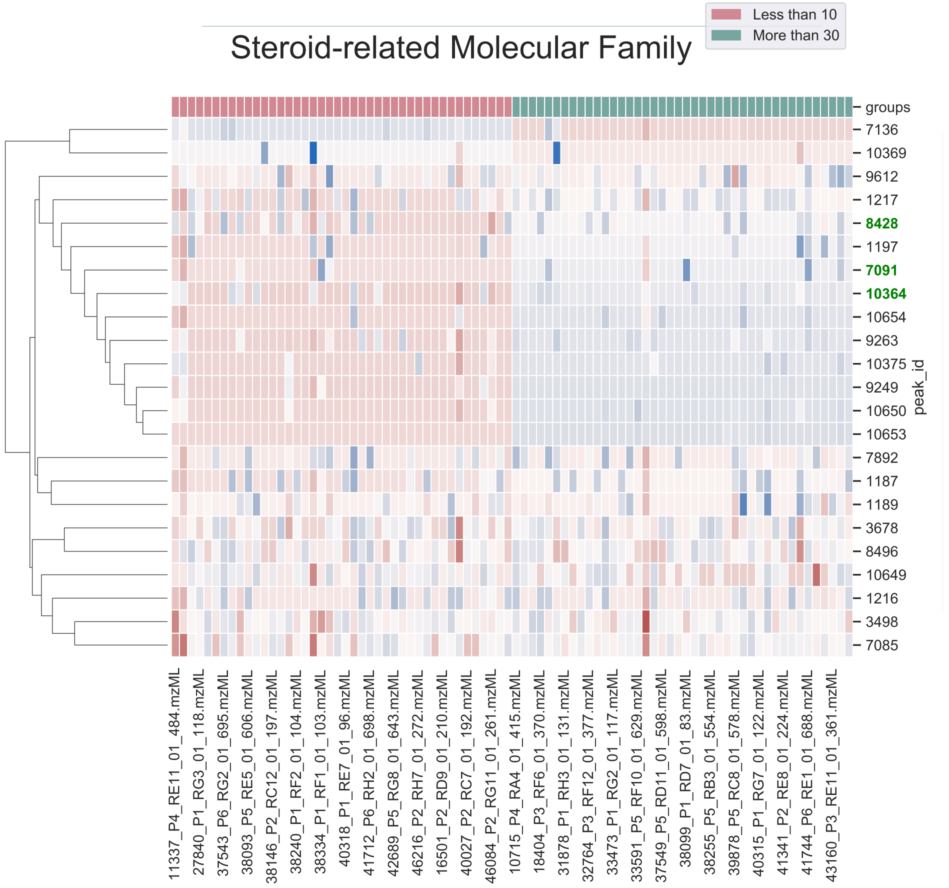

id	LibraryID	m/z	RT	Intensity	no_spectra
1187		357.2056	3.0498	0.0377	61
1189		385.2352	3.4066	0.0058	85
1197		343.2264	3.6391	0.0185	28
1216		367.2269	3.3133	0.0036	86
1217		339.1964	3.0423	0.0038	109
3498		769.4667	3.3526	0.001	174
3678		399.3253	5.7595	0.0008	159
7085		713.4052	3.0489	0.0031	176
7091	Spectral Match to Mestranol from NIST14	311.2008	3.054	0.0012	24
7136		343.2255	3.2039	0.0002	25
7892		385.2367	3.1743	0.0133	68
8428	adrenosterone	301.1801	2.7956	0.0034	98
8496		383.3314	8.3489	0.0076	196
9249		343.2264	3.7812	0.0033	25
9263		369.241	4.2581	0.0005	56
9612		371.2578	4.3831	0.0082	90
10364	Spectral Match to Boldione from NIST14	285.1853	3.1896	0.0009	52
10369		413.3041	5.2675	0.0006	16
10375		313.2165	3.7372	0.0004	28
10649		685.4481	3.7336	0.0011	124
10650		369.2419	4.1228	0.0019	30
10653		341.2097	3.6374	0.0002	19
10654		325.2157	3.6832	0.0004	17

‘ Finally for MS2LDA analysis, the AGP results for the FBMN workflow was further ran through the MS2LDA workflow on GNPS. This GNPS-MS2LDA result is available from `https://gnps.ucsd.edu/ProteoSAFe/status.jsp?task=7c34badae00e43bc87b195a706cf1f43`. The MS1 peak table from the original FBMN result was used for this analysis, as well as the provided metadata CSV. A significantly changing ferulic-acid related motif (p-value $\leq$ 0.001) can be found below.

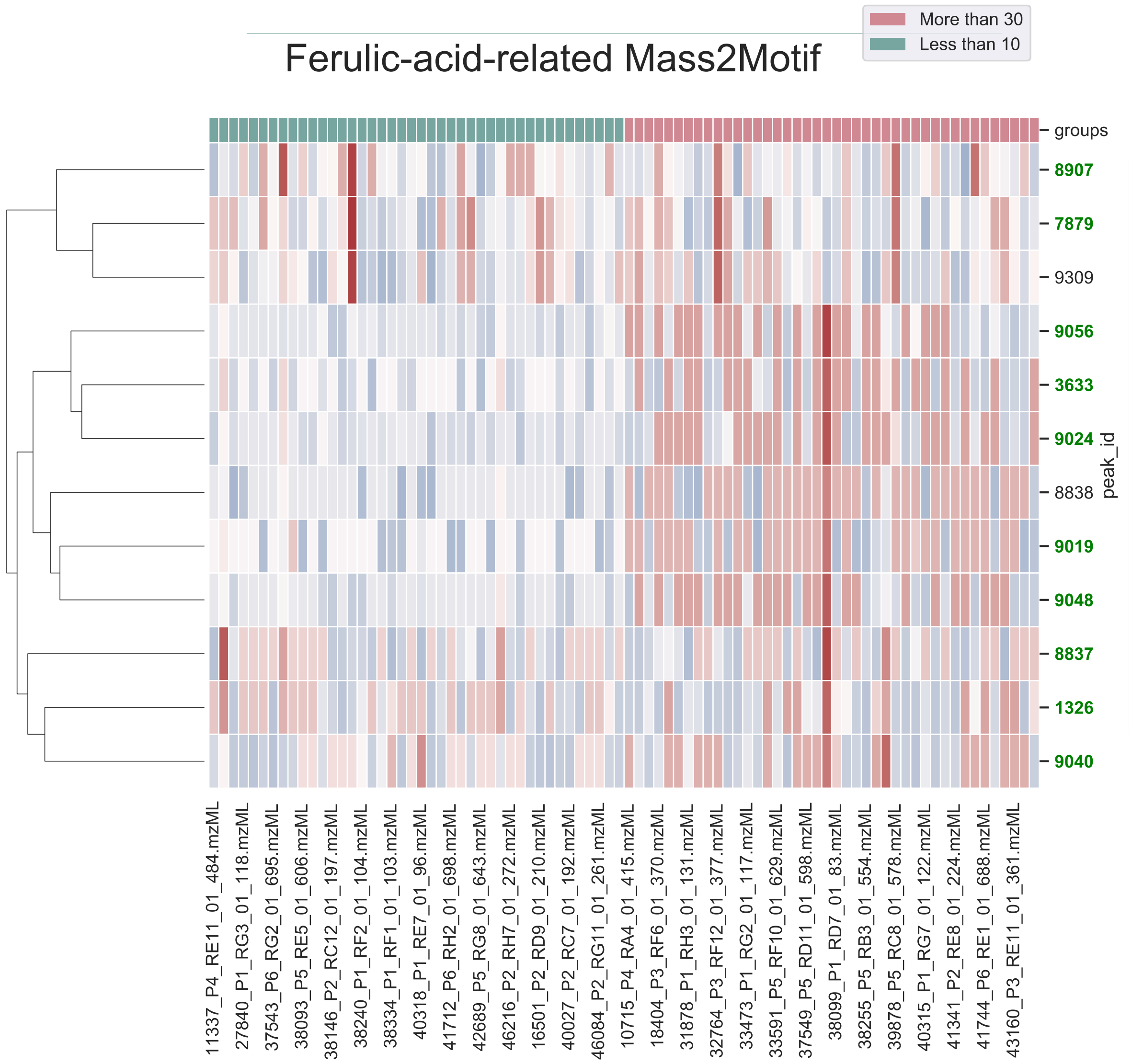

id	LibraryID	m/z	RT	Intensity	no_spectra
1326	Spectral Match to Curcumin from NIST14	369.1335	4.0253	0.0322	145
3633	Spectral Match to Curcumin from NIST14	369.1336	3.1547	0.0024	97
7879	Spectral Match to 3-Hydroxy-4-methoxycinnamic acid from NIST14	195.0655	2.475	0.002	192
8837	Spectral Match to 3-Hydroxy-4-methoxycinnamic acid from NIST14	177.0538	4.0199	0.0005	127
8838		371.149	3.8366	0.0046	49
8907	MoNA:3697220 Feruloyltyramine	265.1549	0.889	0.0015	177
9019	NCGC00095321-06!(1E,4Z,6E)-5-hydroxy-1,7-bis(4-hydroxy-3-methoxyphenyl)hepta-1,4,6-trien-3-one	369.1332	4.275	0.0016	85
9024	(1R,3R,4S,5R)-1,3,4-trihydroxy-5-[(E)-3-(4-hydroxyphenyl)prop-2-enoyl]oxycyclohexane-1-carboxylic acid	339.123	3.147	0.0005	103
9040	Spectral Match to Curcumin from NIST14	369.1331	3.877	0.0031	128
9048	Spectral Match to Curcumin from NIST14	369.1331	4.4907	0.0007	84
9056	NCGC00168971-02_C17H20O9_(1R,3R,4S,5R)-1,3,4-Trihydroxy-5-{[(2E)-3-(4-hydroxy-3-methoxyphenyl)-2-propenoyl]oxy}cyclohexanecarboxylic acid	369.1335	3.3287	0.0003	105
9309		177.0552	2.4861	0.0011	177

Supplementary Section S9: Analysis of Differentially Expressed Mass2Motifs from the Rhamnaceae Dataset

Using PALS Viewer, activity level analysis was run on the results of GNPS-MS2LDA workflow from [1] containing 25 Mass2Motifs that had been manually characterized and their distribution over the Rhamnaceae clades was examined.

The GNPS-MS2LDA data can be found from `https://gnps.ucsd.edu/ProteoSAFe/status.jsp?task=b33b2697e7924ee1920dba207ed57733`. To perform this analysis, users also need to upload the MS1 peak table (`https://github.com/glasgowcompbio/PALS/raw/master/notebooks/test_data/Rhamnaceae/171205_71extracts_MS1peaktable_MS2LDA_comma.csv`, as well as the metadata CSV describing which mzML files belong to which genera (`https://github.com/glasgowcompbio/PALS/raw/master/notebooks/test_data/Rhamnaceae/MetaData_Rhamnaceae.csv`).

We performed a comparison between the Rhamnus (control) and Ziziphus (case) genera. The results, shown in the following table, revealed that Mass2Motifs annotated with flavonoid-related substructures (i.e., rhamnocitrin, kaemfperol, flavonoid core framgent, and emodin) are all differently expressed between the Rhamnus and Ziziphus genera. The results here are consistent with the original study in [1],

**Mass2Motif**	**p-value**	**No. of members**
rhamn_motif_130.m2m [Kaempferol]	0.000000E+00	68
rhamn_motif_121.m2m [CHOOH loss - indicative for underivatized carboxylic acid group]	0.000000E+00	29
rhamn_motif_140.m2m [Flavonoid core fragments (m/z 151)]	0.000000E+00	25
motif_81	0.000000E+00	24
rhamn_motif_141.m2m [Emodin related Motif]	0.000000E+00	21
motif_50	0.000000E+00	12
rhamn_motif_163.m2m [Glycosyl moiety]	4.326859E-66	66
rhamn_motif_40.m2m [Emodin related Motif]	8.907510E-65	18
rhamn_motif_164.m2m [rhamnocitrin-related]	1.215099E-19	17
motif_98	3.335502E-17	11
rhamn_motif_172.m2m [CO2 loss]	3.818233E-16	44
motif_103	3.091902E-13	20
motif_38	1.665557E-12	14
motif_127	9.432444E-12	13
motif_82	4.864967E-11	13
rhamn_motif_28.m2m [coumaric acid-related]	9.965255E-11	23
rhamn_motif_87.m2m [(epi)ceanothic acid-related]	1.021007E-09	27
rhamn_motif_51.m2m [Cyclopeptide alkaloids]	1.164000E-09	16
rhamn_motif_167.m2m [Flavonoid core fragment (m/z 152)]	1.577084E-09	33
rhamn_motif_120.m2m [Coumaric acid - H2O]	1.594465E-09	50
motif_88	1.607455E-09	14
rhamn_motif_179.m2m [Rhamnetin (=7-methylquercetin)]	8.683922E-09	19
motif_79	1.852689E-08	21
rhamn_motif_153.m2m [CO2 loss]	4.288013E-08	39
rhamn_motif_169.m2m [coumaric acid related]	5.868700E-08	11
rhamn_motif_108.m2m [CHOOH loss - indicative for underivatized carboxylic acid group]	1.169172E-07	15
rhamn_motif_60.m2m [CO2/H2O loss]	1.387670E-07	26
motif_72	4.529178E-07	16
rhamn_motif_165.m2m [ceanothic acid A-ring CO2 loss]	1.318498E-06	24
rhamn_motif_33.m2m [Xyl or Ara moiety]	3.280194E-06	25
motif_84	6.478872E-06	11
motif_125	1.197932E-05	12
motif_44	1.593861E-05	83
rhamn_motif_48.m2m [Cyclopeptide alkaloids]	2.034794E-05	12
rhamn_motif_148.m2m [Cyclopeptide alkaloids]	2.206457E-05	25
motif_107	6.337228E-05	11
rhamn_motif_117.m2m [protocatechuoyl-related]	9.157846E-05	19
motif_92	1.285183E-04	31
motif_74	1.361637E-04	17
rhamn_motif_191.m2m [vanilloyl-related]	2.172679E-04	25
motif_75	2.500638E-04	32
rhamn_motif_34.m2m [Sugar (Glc) Loss]	2.780931E-04	29
motif_106	4.134637E-03	13
motif_120	4.561325E-03	14
rhamn_motif_64.m2m [Norrubrofusarin-related]	5.617434E-03	13

References

[1] Kang, K. B. *et al.* (2019). Comprehensive mass spectrometry-guided phenotyping of plant specialized metabolites reveals metabolic diversity in the cosmopolitan plant family rhamnaceae. *The Plant Journal*, **98**(6), 1134–1144.

[2] Khatri, P. *et al.* (2012). Ten years of pathway analysis: current approaches and outstanding challenges. *PLoS Computational Biology*, **8**(2), e1002375.

[3] McDonald, D. *et al.* (2018). American gut: an open platform for citizen science microbiome research. *MSystems*, **3**(3), e00031–18.

[4] Nothias, L. F. *et al.* (2019). Feature-based molecular networking in the GNPS analysis environment. *bioRxiv*, page 812404.

[5] Subramanian, A. *et al.* (2005). Gene set enrichment analysis: a knowledge-based approach for interpreting genome-wide expression profiles. *Proceedings of the National Academy of Sciences of the United States of America*, **102**(43), 15545–15550.
